# Novel compound heterozygous SIL1 variants associated with Marinesco-Sjögren syndrome in a Chinese family

**DOI:** 10.3389/fgene.2026.1795147

**Published:** 2026-08-28

**Authors:** Yizheng Jiang, Aojie Lian, Jialing Guo

**Affiliations:** 1 NHC Key Laboratory of Birth Defect for Research and Prevention, Hunan Provincial Maternal and Child Health care Hospital, Changsha, Hunan, China; 2 School of Life Sciences, Central South University, Changsha, Hunan, China; 3 Hunan Provincial University Key Laboratory of the Fundamental and Clinical Research on Neurodegenerative Diseases, Changsha Medical University, Changsha, Hunan, China

**Keywords:** compound heterozygous variants, gene, Marinesco-Sjögren syndrome, SIL1, whole exome sequencing

## Abstract

**Background:**

Marinesco-Sjögren syndrome (MSS) is a rare and disabling genetic disorder caused primarily by pathogenic variants in the SIL1 gene. SIL1 functions as a nucleotide exchange factor for the molecular chaperone BiP within the endoplasmic reticulum (ER), which is essential for protein folding. This study aims to characterize a novel SIL1 compound heterozygous pathogenic mutation and investigate its disease-causing mechanism.

**Methods:**

We determined a novel SIL1 compound heterozygous mutation information using whole exome sequencing and Sanger sequencing. RNA-seq, RT-qPCR and Western blot were employed to assess the impact of mutations on SIL1 RNA and protein levels. Immunofluorescence was used to monitor localization changes at the cellular level. Structural prediction and coimmunoprecipitation were utilized to investigate the effects of mutations on protein interactions.

**Results:**

We identified a 6-year-old girl with MSS carrying novel compound heterozygous variants in the SIL1 gene (c.570_572delCAA (p. Asn190del) and c.740C>T (p. Ala247Val)). Subsequent functional experiments revealed that the mutations show a reduction in SIL1 mRNA and protein levels. Structural prediction and coimmunoprecipitation indicate that the compound variants may be associated with the development of MSS by weakening SIL1-BiP binding.

**Conclusions:**

These findings expand the SIL1 variant spectrum and provide preliminary functional evidence that the identified variants may affect SIL1 abundance and SIL1-BiP interaction, supporting their relevance to the MSS phenotype in this family.

## Background

Marinesco-Sjögren syndrome (MSS; OMIM 248800) is a debilitating autosomal recessive condition characterized by a constellation of clinical features, including cerebellar ataxia, congenital or early-onset cataracts, progressive myopathy, intellectual disability, and short stature (Marinesco, 1931; Sewry et al., 1988). Additional, less consistent features can include skeletal abnormalities and hypergonadotropic hypogonadism. Initial genetic investigations mapped a locus for MSS to chromosome 5q31, but the underlying causative gene remained elusive (Lagier-Tourenne et al., 2003). Although a mutation in the SAR1B gene, located within this critical interval, was identified in a single family presenting with a unique combination of MSS and chylomicron retention disease, this gene was not found to be responsible for classical MSS (Jones et al., 2003). Furthermore, the genetic heterogeneity of MSS-like disorders was highlighted by the discovery that mutations in CTDP1 on chromosome 18qter cause a clinically related syndrome, a possibility that was excluded in typical MSS cohorts (Varon et al., 2003). This complex genetic landscape underscored the need for a definitive molecular diagnosis for the majority of MSS cases.

Subsequent research has now firmly established that mutations in the SIL1 gene are the primary cause of Marinesco-Sjögren syndrome. SIL1 (also known as BAP) is an essential resident protein of the endoplasmic reticulum (ER) that functions as a co-chaperone. Specifically, it acts as a nucleotide exchange factor for the highly conserved Hsp70 chaperone BiP (also called GRP78), promoting the exchange of ADP for ATP to regulate BiP’s activity cycle (Chung et al., 2002). The identification of multiple distinct, disruptive mutations in SIL1 across several families with MSS provides compelling evidence for its pathogenic role (Senderek et al., 2005).

The discovery of SIL1 as the causative gene for MSS implicates ER dysfunction as the core cellular pathology of the disease. The BiP chaperone, regulated by SIL1, plays a pivotal role in two fundamental ER processes: facilitating the translocation of newly synthesized proteins into the ER lumen and overseeing their subsequent folding and quality control (Schnell and Hebert, 2003; Alder et al., 2005; Hendershot, 2004). Therefore, a loss of SIL1 function is predicted to severely compromise these activities. This impairment can lead to a cascade of detrimental cellular events, including reduced synthesis of critical proteins, accumulation of unfolded or misfolded proteins, and the induction of the unfolded protein response (UPR), a major cellular stress pathway that can ultimately trigger apoptosis (Rao et al., 2004). By framing MSS as a disorder of ER protein homeostasis, these findings provide a crucial molecular entry point for understanding the developmental and degenerative processes affecting the cerebellum, lens, and skeletal muscle in this complex multisystem disease (Suzuki et al., 1997; Kitao et al., 2004; Tanaka et al., 1998).

In this study, we identified a MSS patient with a novel SIL1 compound heterozygous variants (c.570_572delCAA and c.740C > T) through whole exome sequencing (WES). Both parents carried a single mutation each and exhibited normal phenotypes. Functional experiments showed reduced SIL1 mRNA and protein levels in patient-derived B-LCLs and suggested that the combined variants may weaken SIL1-BiP binding. These findings support the potential biological relevance of the variants, while the specific mechanism remains to be further investigated.

## Methods

### Subjects and clinical examination

The subject of this study was a patient with sporadic Marinesco-Sjögren syndrome (MSS) and her parents. Since both parents were clinically asymptomatic, a comprehensive clinical examination was performed only on the proband. Detailed clinical assessments were conducted at Xiangya Hospital of Central South University, which further confirmed the characteristic clinical manifestations of MSS. Brain MRI was performed on a 3.0 T S Prisma MRI scanner equipped with a standard 32-channel head coil at Xiangya Hospital of Central South University. The control subject was recruited from Hunan Provincial Maternal and Child HealthCare Hospital and had no history of abnormal development, no neurological diseases, and no clinical manifestations similar symptoms related to this patient.

### Whole exome sequencing

The gDNA were analyzed through WES. Briefly, the library was captured using the Illumina Nextera Rapid Capture Exome v1.2 kit and sequenced on Illumina HiSeq 4000 sequencing system. The reads were then aligned to the human genome assembly GRCh38/hg38 using bwa 0.7.17 (Li and Durbin, 2010). Variants were called with GATK 4.6.2.0 (Van der Auwera et al., 2013) and annotated using ANNOVAR (Wang et al., 2010). After filtering out variants with allele frequency >0.01 from databases such as gnomAD and 1000 Genomes Project, all patients shared non-synonymous variants (including single nucleotide variants (SNVs) and insertion/deletion polymorphisms (indels)) in the consensus coding sequence, and the canonical splicing sites were reserved.

### Establishment of B lymphoblastoid cell lines

Briefly, peripheral blood was collected by venipuncture, thoroughly mixed with Roswell Park Memorial Institute (RPMI 1640) medium (Cat: 11875093, Gibco), and added to Histopaque-1077 (Cat: 10771, Sigma). After centrifugation, the lymphocyte layer was aspirated and cultured in a T25 flask with pre-prepared EBV and Ciclosporin A at 37 °C in a humidified incubator containing 5% carbon dioxide. B lymphoblastoid cell lines (B-LCLs) were cultured in RPMI 1640 medium with 25% fetal bovine serum (FBS) (Cat: 10099141, Gibco).

### Primer design

Primers were designed using Primer3 (https://primer3.ut.ee/) based on the human GRCh38/hg38 assemblies. Detailed information on all primers was listed in Supplementary Table S5. The presence of SIL1 mutations was confirmed in MSS family through PCR and Sanger sequencing.

### RNA-sequencing and data analysis

Total RNA was extracted from B-LCLs using TRIzol (Cat: AM9738, Invitrogen) depending on the manufacturer’s instructions. mRNA was enriched using Oligo dT magnetic beads. Following fragmentation, first- and second-strand cDNA was synthesized using random primers. Subsequently, non-strand specific library and strand specific library were performed. After library quality control, Illumina sequencing was performed. SOAPnuke v2.1.9 (Chen et al., 2018) was used for data filtering, and Hisat2 v2.2.1 (Kim et al., 2015) was used to build the reference genome index. The edgeR R package (version 4.6.3) (Chen et al., 2025) was used to perform differential expression analysis. The threshold for significantly differential expression: Padj ≤ 0.05 and |log2(foldchange)| ≥ 1. DisGeNET, GO and KEGG enrichment analysis of differentially expressed genes was implemented by the clusterProfiler 4.16.0 (Yu et al., 2012; Wu et al., 2021), in which gene length bias was corrected.

### RT-qPCR

RT-qPCR experiments were performed using the RevertAid First Strand cDNA Synthesis Kit (Cat: K1622, Thermo Fisher Scientific) and the Maxima SYBR Green qPCR Master Mix (Cat: K0251, Thermo Fisher Scientific) on the CFX96 Real-Time PCR Detection System (Bio-Rad, United States). Beta-Actin (β-Actin) served as the internal controls and data were processed using CFX manager 3.1 (Bio-Rad, United States).

### Western blotting

Samples were lysed in SDS lysis buffer with protease inhibitors (Cat: RM02916, ABclonal). Proteins were separated by SDS-PAGE, and transferred to PVDF membrane. The membranes were incubated with a primary antibody. The primary antibodies used were SIL1 antibody (Cat: ab228844, Abcam) and β-Actin antibody (Cat: A2228, Sigma). Membranes were incubated with HRP-conjugated secondary antibody (Cat: 7074, CST) at room temperature. The protein signals were visualized using the SuperKine West Femto Maximum Sensitivity Substrate (Cat: BMU102, Abbkine) and digitally quantified with the Quantity One (Bio-Rad, United States).

### Cell culture

HEK293T and Hela cells were cultured in DMEM (Cat: C11995500BT, Gibco) containing 10% fetal bovine serum (FBS) (Cat: F2442, Sigma) and 1% penicillin and streptomycin (Cat: 15140122, Thermo Fisher Scientific). Cells were maintained in a 37 °C incubator with 5% CO2.

### Cell cycle and cell apoptosis assay

The Cell Cycle Analysis Kit (Cat: C1052, Beyotime) and the Annexin V-FITC/PI Apoptosis Detection Kit (Cat: A211-01, Vazyme) were used to perform cell cycle and cell apoptosis depending on the manufacturer’s instructions. The assay was conducted on the DXP Athena Flow Cytometer (Cytek, United States), followed by analysis in FlowJo v10.

### Plasmid construction and transfection

The cDNA encoding human SIL1 (NM_001037633) was cloned into the PCAGGS vector, with an additional HA tag added. The cDNA encoding human BiP (NM_005347) was cloned into the PCDNA3.1 vector, with an additional Flag tag added. The plasmid was then transformed into the DH5α. The SIL1 WT, SIL1 p. N190del and SIL1 p. A247V plasmids were obtained using the FastPure Plasmid Mini Kit (Cat: DC221-01, Vazyme). HEK293T and Hela cells were transfected with Lipofectamine 3000 reagent (Cat: 2399133, Thermo Fisher Scientific) according to the manufacturer’s instructions. Transfection efficiency was assessed by fluorescence microscopy 48 h post-transfection.

### Immunofluorescence

The Hela cells were washed with 1× PBS, fixed with 4% PFA, and permeabilized with 0.1% Triton-X in 1× PBS at room temperature. The sections were then blocked with 5% bovine serum albumin (BSA) in 1× PBS containing 0.3M glycine and incubated overnight with a primary antibody diluted with 5% BSA in 1× PBS at 4 °C. After incubation with a fluorescent-conjugated second antibody and the nuclear staining with 4′,6-Diamidino-2-phenylindole dihydrochloride (DAPI), the sections were mounted and captured using a confocal microscope (Leica TCS SP5, Leica Biosystems). Colocalization was analyzed using ImageJ software. The primary antibodies used were anti-HA (Cat: 51064-2-AP, Proteintech) and anti-BiP (Cat: A27312, Abclonal).

### Protein structure analysis

AlphaFold3 (version 3.0.1) and AlphaFold Multimer structural prediction models were employed to predict the protein structures of single and double mutants and their complexes. Model construction and image refinement were performed and completed in PyMOL (version 3.0) and DynaMut.

### Coimmunoprecipitation

Cells were lysed using lysis buffer containing protease inhibitors. After shaking at 4 °C for 30 min, the sample were centrifuged, and the supernatant was collected as the Input group. The supernatant was incubated with HA magnetic beads (Cat: HY-K0201, MCE) overnight at 4 °C. After washing the magnetic beads, the proteins were eluted, and finally Western blot analysis was performed on the Input and immunoprecipitation (IP) groups. The antibodies used were anti-HA and anti-Flag (Cat: 20543-1-AP, Proteintech). For quantification, the coimmunoprecipitated Flag-BiP signal in each IP sample was normalized to the corresponding immunoprecipitated HA-SIL1 signal (Flag/HA ratio) to account for differences in SIL1 expression and immunoprecipitation efficiency among constructs. Normalized values were then expressed relative to the WT SIL1 group.

### Statistical analysis

For RNA-seq, one B-LCL sample was analyzed for each individual (normal control, I:1, I:2 and II:1). For RT-qPCR, Western blotting, flow cytometry, immunofluorescence and coimmunoprecipitation assays, n = 3 refers to independent experimental repeats. The data were presented as mean ± SEM. Significances were calculated using one-way analysis of variance (ANOVA), with a p-value <0.05 considered significant difference.

## Results

### Clinical characteristics

**FIGURE 1:**
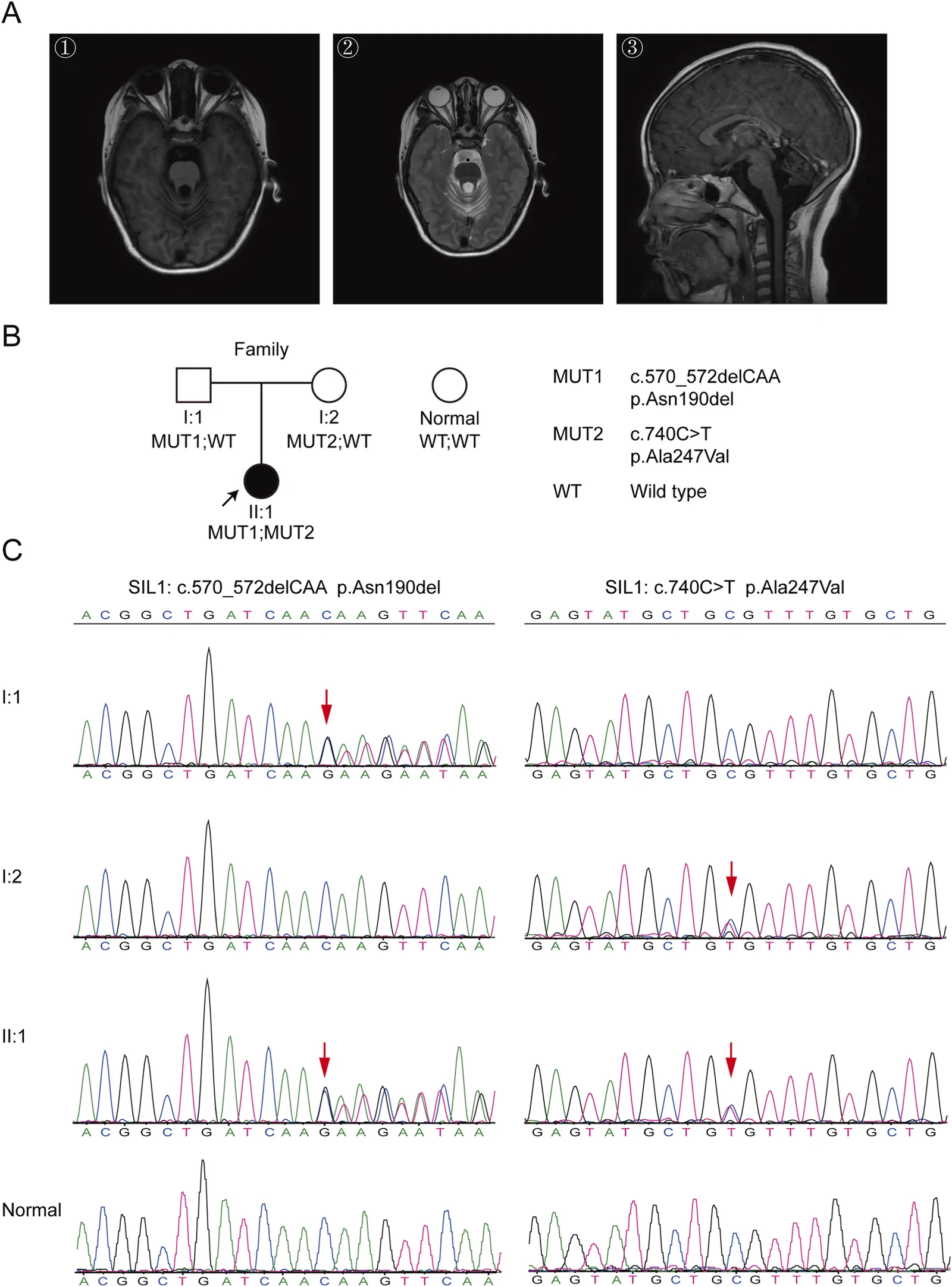
Imaging features and genetic studies of the Marinesco-Sjögren syndrome (MSS) family. **(A)** MRI manifestations of the affected individual II:1 from the MSS family. MRI of brain showing: ①Enlarged fourth ventricle. ②Cerebellar atrophy, particularly in the vermis. ③Enlarged subarachnoid space attributable to cerebellar atrophy. **(B)** Pedigree of the MSS family. II-1 is the proband, and she carries compound heterozygous mutations, while her parents carry a single heterozygous mutation. Normal individuals do not carry the mutation. **(C)** Gene mutation analysis on the MSS family and normal individuals by Sanger sequencing.

**FIGURE 2:**
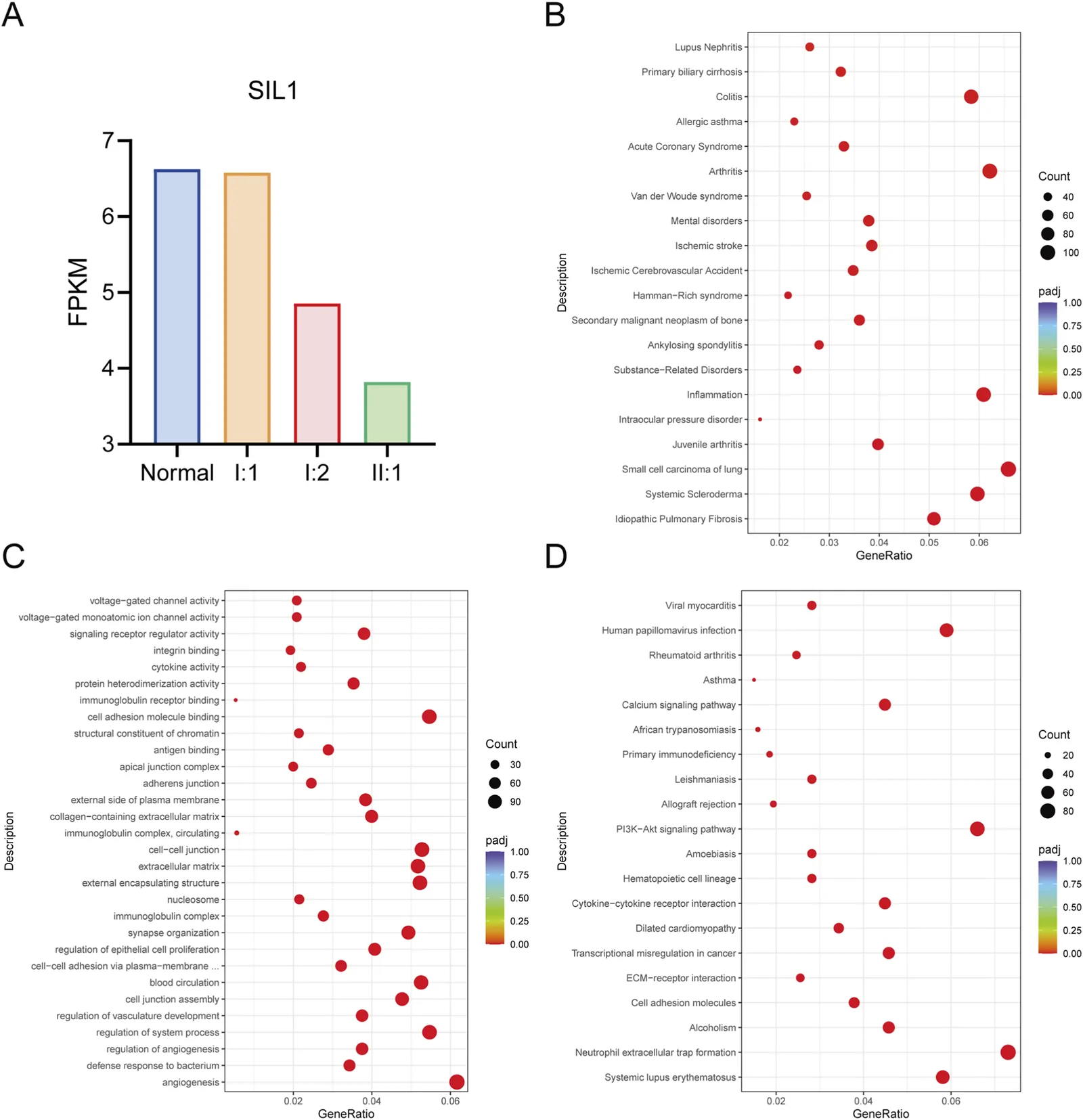
Differential expression and functional analysis of mRNAs. **(A)** Expression levels of SIL1 in the B-LCLs from normal, (I)1, (I)2 and II:1. n = 1 for each individual. **(B)** DisGeNET analysis of differentially expressed mRNAs (II-1 vs. Normal). **(C)** GO enrichment analysis of differentially expressed mRNAs (II-1 vs. Normal). **(D)** KEGG enrichment analysis of differentially expressed mRNAs (II-1 vs. Normal). Genes with adjusted P-value≤0.05 were considered as differentially expressed genes. Pathways with P-value of≤0.05 were considered as significantly enriched pathways.

**FIGURE 3:**
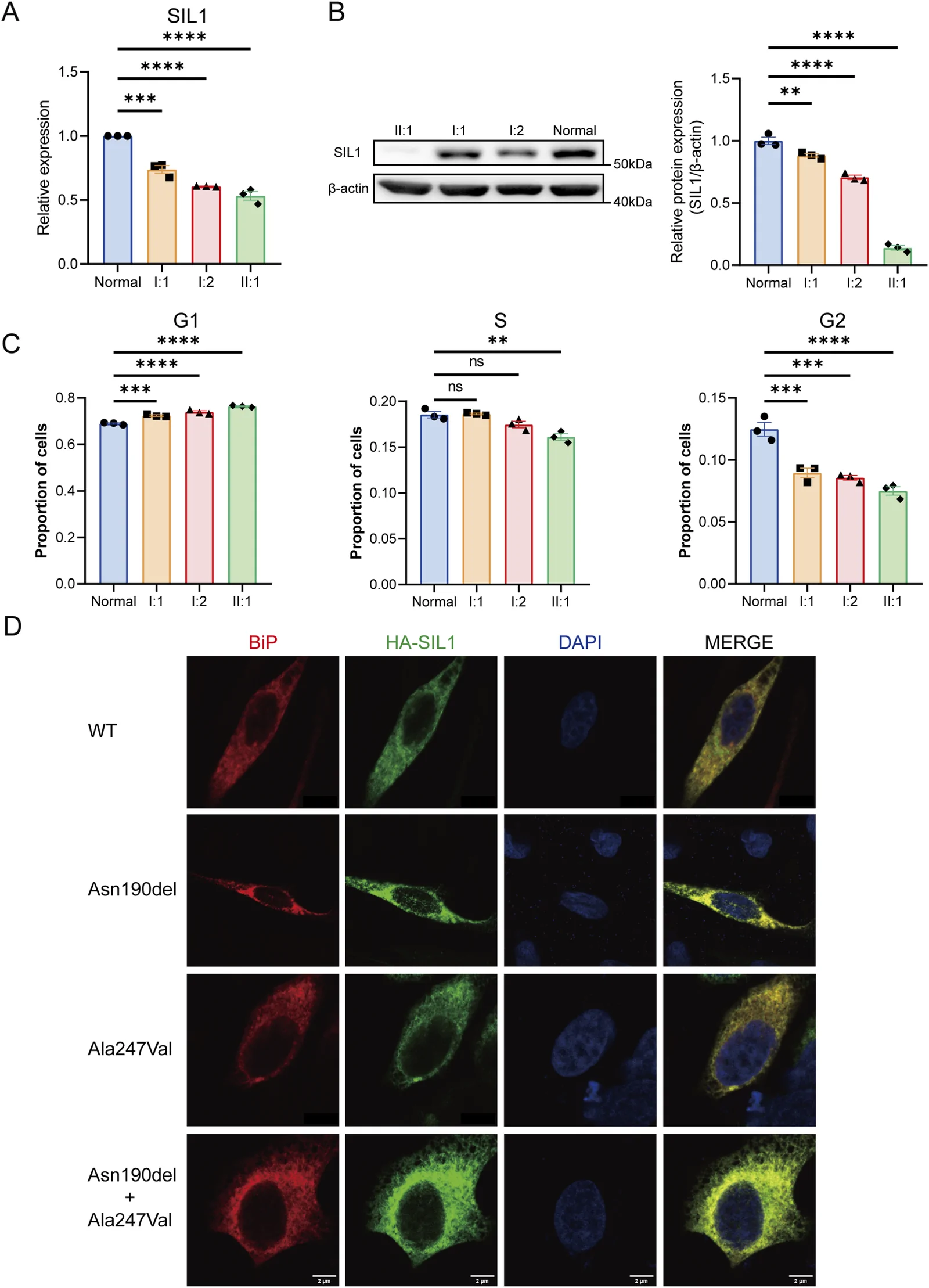
The expression, cell cycle and localization of SIL1 mutants. **(A)** RT-qPCR detection of SIL1 mRNA expression in the B-LCLs from normal, (I)1, (I)2 and II:1. n = 3 independent experimental repeats. Statistic data were analyzed using one-way ANOVA. **(B)** Western blot analysis of SIL1 in the B-LCLs from normal, (I)1, (I)2 and II:1. And relative protein quantification of grey scale value for SIL1 and β-actin. n = 3 independent experimental repeats. Statistic data were analyzed using one-way ANOVA. **(C)** Cell cycle analysis of B-LCLs from normal, (I)1, (I)2 and II:1. n = 3 independent experimental repeats. Statistic data were analyzed using one-way ANOVA. **(D)** Immunofluorescent staining of wild type and mutated plasmids overexpressed in Hela cells. Scale bar = 2 μm.

The proband is a six-year-old female who was born at term via cesarean delivery, with no history of birth asphyxia, trauma, or resuscitation. She began to exhibit developmental delays from the age of three months, initially presenting with poor head control. As she grew older, her language, cognitive, and motor skills consistently lagged behind those of her peers. At five years of age, she was able to communicate using short phrases but exhibited dysarthria. By the age of 6, she could walk independently for approximately 30 steps, though with significant instability, uncoordinated movements, and gait ataxia. No visual or hearing impairments were observed, and there was no history of seizures. MRI of her brain showing: Enlarged fourth ventricle (Figure 1A-①), Cerebellar atrophy, particularly in the vermis (Figure 1A-②) and enlarged subarachnoid space attributable to cerebellar atrophy (Figure 1A-③). Physical examination revealed: height between the 10th and 25th percentiles, weight above the 97th percentile, intellectual disability, nystagmus, generalized hypotonia, ataxic gait, and a positive Romberg’s sign. Both parents of the proband are phenotypically normal. The clinical characteristics were summarized in Supplementary Table S1.

### Genetic testing results

We collected peripheral blood samples from the proband and her unaffected parents, extracted DNA samples, and performed WES. We detected a heterozygous in-frame deletion mutation in the SIL1 gene [NM_001037633.2: c.570_572delCAA (p. Asn190del)] and a heterozygous missense mutation [NM_001037633.2: c.740C>T (p. Ala247Val)] in the proband (II:1). The I:1 (the proband‘s father) carries the heterozygous variant c.570_572delCAA (p. Asn190del) but not c.740C>T (p. Ala247Val) variant, whereas the I:2 (the proband‘s mother) possesses the heterozygous variant c.740C>T (p. Ala247Val) without the c.570_572delCAA (p. Asn190del) variant. And neither mutation was present in the normal control sample (Figure 1B). Subsequent sanger sequencing validated the aforementioned findings (Figure 1C). No additional variants capable of explaining the patient’s clinical symptoms were detected, including mitochondrial genome variants or potential copy number variations.

By querying population databases such as ExAC and GnomAD, both the SIL1 mutations were identified as rare variants (Supplementary Table S2). And according to the ACMG guidelines and ClinGen SVI expert group’s recommendations (Biesecker et al., 2018; Abou Tayoun et al., 2018; Ghosh et al., 2018), both mutations are classified as variants of uncertain significance [c.570_572delCAA (p. Asn190del): PM2 + PM4_supporting evidence; c.740C > T (p. Ala247Val): PM2 + PP1_supporting evidence].

The c.570_572delCAA (p. Asn190del) variant has not been previously reported, whereas the c.740C > T (p. Ala247Val) mutation has been associated with relevant findings. Related study identified SIL1: c.740C > T (p. Ala247Val) and c.783G > C (p. Gln261His) compound heterozygous mutations in two patients with cerebellar ataxia and dysarthria, whose parents each carried one mutation but exhibited no clinical symptoms (Noreau et al., 2015). We analyzed the mutations using the functional prediction tools in the VarCards2 database (Wang et al., 2024). The results indicate that the variants c.570_572delCAA (p. Asn190del) and c.740C > T (p. Ala247Val) are not pathogenic (Supplementary Table S3).

### Compound heterozygous variants dramatically decrease expression of SIL1 gene

To further investigate the molecular mechanism underlying composite heterozygous pathogenicity, we established B lymphoblastoid cell lines (B-LCLs) from collected human peripheral blood samples through Epstein Barr virus (EBV) infection. And RNA sequencing (RNA-seq) was performed using B-LCLs from the normal control, I:1, I:2 and II:1, with one sample per individual. As shown in Figure 2A, SIL1 expression was lower in the three family members than in the normal control, with the lowest level observed in the proband (II:1). Quantitative PCR (RT-qPCR) analysis further supported the reduction of SIL1 expression (Figure 3A). We then performed an exploratory comparison between II:1 and the normal control using DisGeNET, Gene Ontology (GO) and Kyoto Encyclopedia of Genes and Genomes (KEGG) pathway analyses to investigate the potential function of SIL1 compound heterozygous mutation-associated mRNAs. It was found that the SIL1 compound heterozygous mutations c.570_572delCAA (p. Asn190del)- and c.740C>T (p. Ala247Val)-associated mRNAs are significantly enriched in terms of mental disorders and synapse organization, with the most affected pathways including the PI3K-Akt signaling pathway and Calcium signaling pathway (Figures 2B–D). However, since the RNA-seq was performed on B-LCLs, which is not the MSS-affected tissue, and because no biological replicates were available, these RNA-seq and enrichment results should be interpreted as hypothesis-generating evidence, and future studies are needed to further validate these discoveries.

### Protein expression and subcellular localization analysis of the SIL1 variants

To determine whether the variants affect SIL1 protein expression, we performed Western blot analysis in B-LCLs from MSS family and normal. Consistent with the trend observed in mRNA, SIL1 protein expression was reduced in all three individuals from the MSS family compared to normal, with the most significant decrease observed in II-1 (Figure 3B). Cell cycle analysis revealed an increase in the G1 phase and a decrease in the S and G2 phases for II-1 compared to normal (Figure 3C). In the apoptosis assay, early apoptosis was higher in II-1 compared to the normal, with no significant changes in late apoptosis (Supplementary Figure S1A). To assess subcellular localization, we constructed SIL1 wild type (WT) and mutant plasmids for transfection into Hela cells. The results indicate that, compared to the wild-type, SIL1 tends to colocalize with BiP (Endoplasmic reticulum marker) regardless of whether it carries a single mutation or a compound heterozygous mutation (Figure 3D; Supplementary Figure S1B).

### Homology and protein structural analysis of the SIL1 variants

**FIGURE 4:**
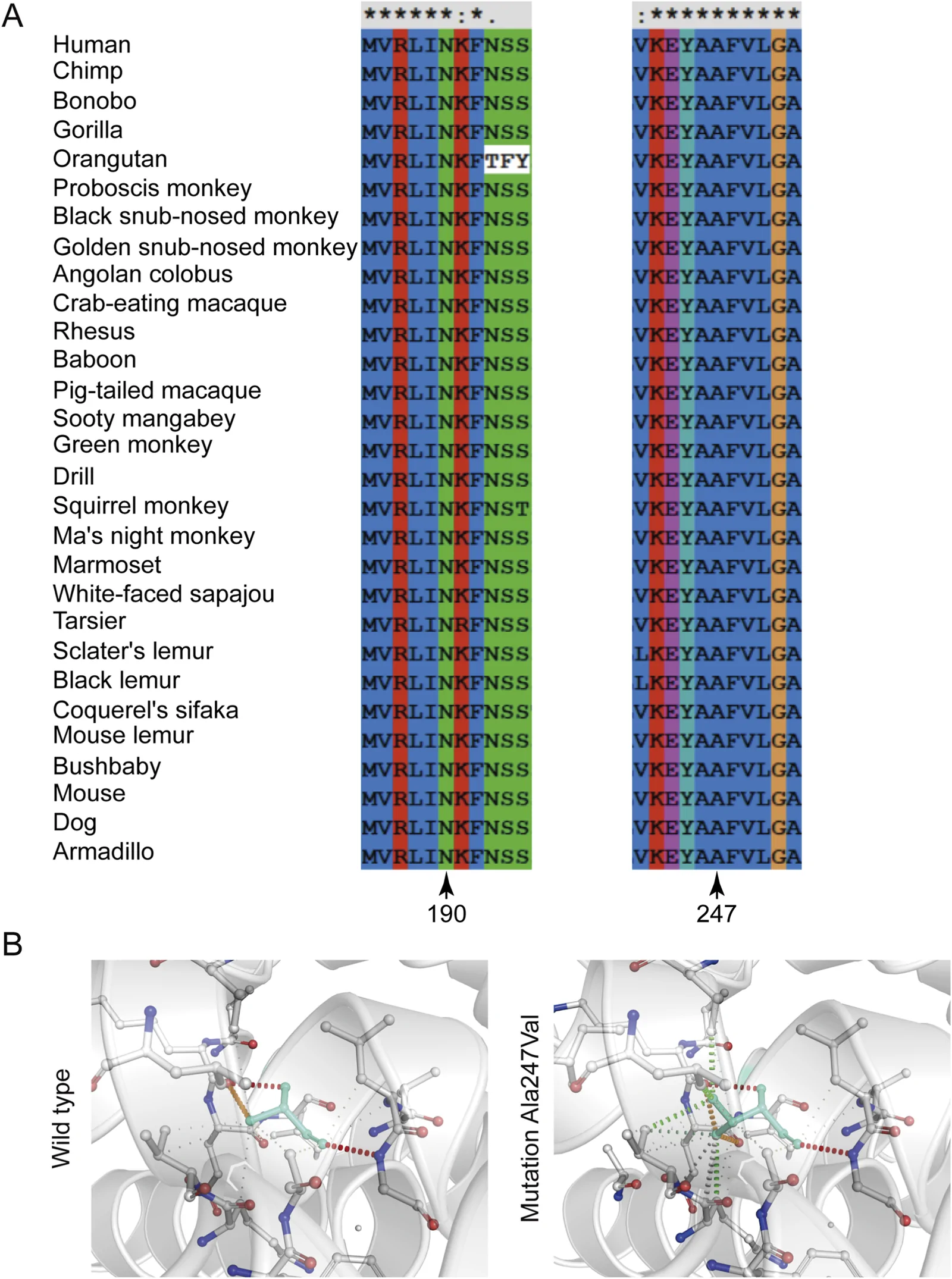
Homology and structural characterization analysis of SIL1 mutants. **(A)** Amino acid conservation alignment of Asn190 and Ala247. **(B)** Comparison of structural changes between wild type and Ala247Val variant in SIL1. Wild type and mutant residues are colored in light-green and are also represented as sticks alongside with the surrounding residues which are involved on any type of interactions. The green dashed lines indicate hydrophobic contacts and the red dashed lines represent hydrogen bonds between amino acids.

Homology analysis was conducted on two mutation sites of the SIL1 gene. Amino acids p. Asn190 and p. Ala247 of the SIL1 gene were highly conserved in vertebrates (Figure 4A). This suggests a potential functional relevance. We also assessed the effects of the c.740C>T (p. Ala247Val) mutation on protein stability and dynamics using DynaMut (Rodrigues et al., 2018). Prediction results indicate that the p. Ala247Val mutation may induce destabilizing effects on the overall protein structure and reduce molecular flexibility (Supplementary Figure S2; Supplementary Table S4). From the perspective of bond formation, the p. Ala247Val mutation may increase the number of molecules involved in hydrophobic interactions and the number of hydrogen bonds within the protein structure (Figure 4B). The structural predictions above indicate a modest potential impact of the p. Ala247Val substitution on protein stability, although the magnitude of this effect was limited.

**FIGURE 5:**
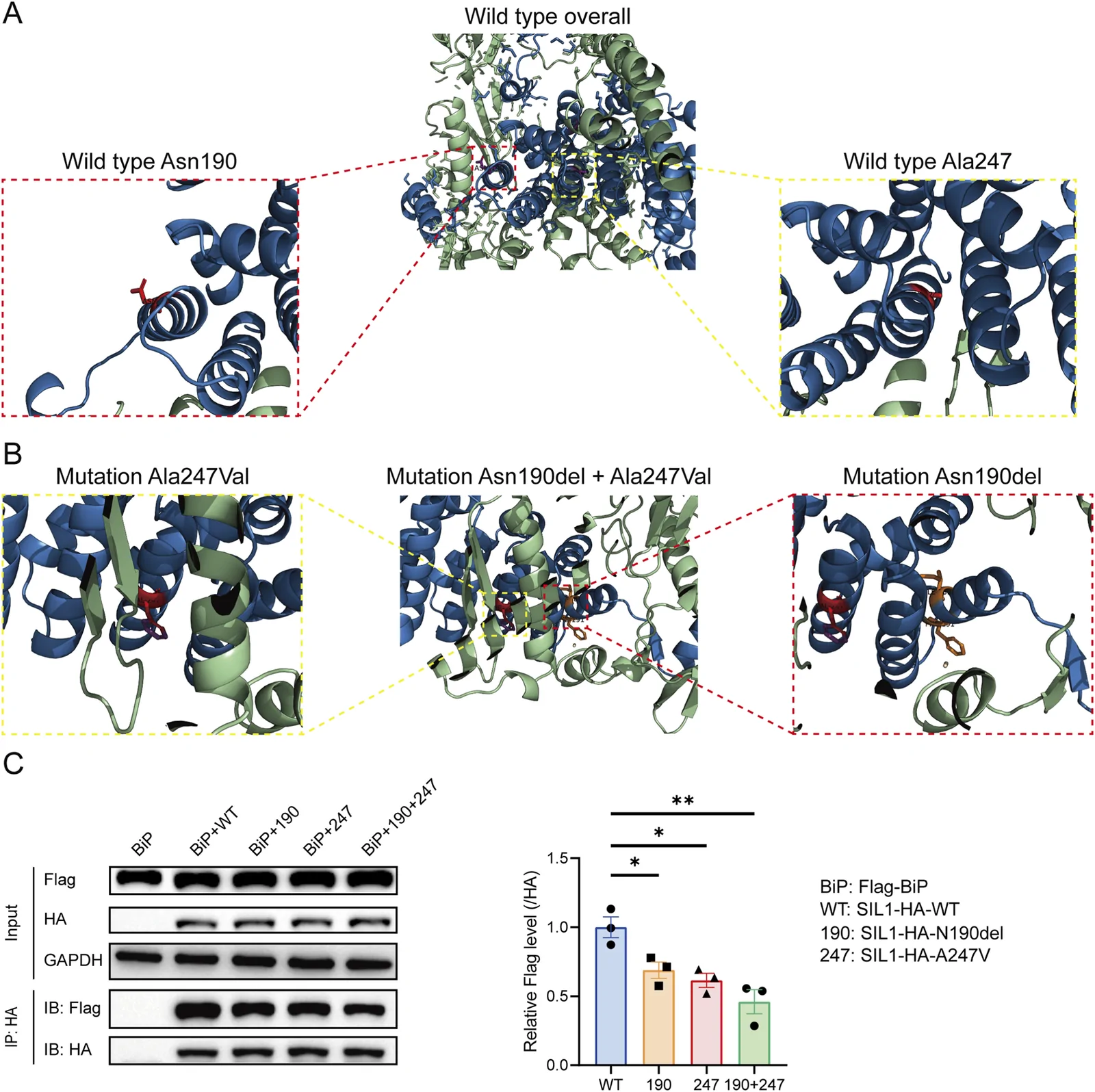
SIL1 variants may weaken protein interaction with BiP. **(A)** Overall view of Asn190 and Ala247 in the predicted SIL1-BiP protein structure. **(B)** View of the Asn190del and Ala247Val compound mutant in the SIL1-BiP interacting protein structure. In the image, blue chains represent SIL1, green chains represent BiP, red indicates mutated amino acid residues, yellow denotes hydrogen bonds, wheat signifies salt bridges, and deep purple represents electrostatic interactions. **(C)** Coimmunoprecipitation assay for SIL1 and BiP in HEK293T cells. The coimmunoprecipitated Flag-BiP signal in each IP sample was normalized to the corresponding immunoprecipitated HA-SIL1 signal (Flag/HA ratio) to account for potential differences in SIL1 expression and immunoprecipitation efficiency among constructs, and normalized values were expressed relative to the WT SIL1 group. n = 3 independent experimental repeats. Statistic data were analyzed using one-way ANOVA.

To investigate the potential mechanism associated with the SIL1 compound heterozygous variants, we performed structural analysis using AlphaFold3 and PyMOL (Abramson et al., 2024; Mooers and Brown, 2020). The prediction results suggest possible alterations at the predicted SIL1-BiP interaction interface for the p. Asn190del and p. Ala247Val combination. However, these observations are based solely on computational predictions and require experimental validation to determine their biological significance (Figures 5A,B). We therefore performed coimmunoprecipitation assays for SIL1 and BiP. Compared with WT SIL1, the mutant SIL1 constructs showed reduced BiP co-IP signal, with the p. Asn190del + p. Ala247Val construct showing the most pronounced reduction (Figure 5C). These results support a potential weakening of SIL1-BiP binding by the variants.

## Discussion

In this study, we identified a novel compound heterozygous mutation of SIL1 that was composed of a paternally inherited c.570_572delCAA (p. Asn190del) variation and a maternally inherited c.740C > T (p. Ala247Val) variation and was closely associated with MSS in a Chinese family. To investigate the potential biological effects of these variants, we successfully established B-LCLs by infecting peripheral blood lymphocytes from MSS family with EBV. Functional assay results indicate that compared to normal, B-LCLs carrying the c.570_572delCAA variant showed reduced SIL1 mRNA and protein levels. The c.740C > T (p. Ala247Val) mutation exhibits a greater degree of reduction, while the compound mutation of both variants demonstrates the most pronounced decrease. Interestingly, parents carrying a single mutation exhibit a normal phenotype. Therefore, we propose this pattern is consistent with a possible dose-related effect, but does not prove a definitive dose-dependent model. And since this study is based on only one family, we hope that this explanation can be validated through other cases or disease-related models.

Through a review of relevant papers and databases, the c.570_572delCAA (p. Asn190del) mutation has not been reported, while the c.740C > T (p. Ala247Val) mutation was identified in a family with cerebellar ataxia and atrophy (Noreau et al., 2015). Both affected individuals in this family carry the same SIL1 compound heterozygous mutation, which consists of a paternal c.740C > T (p. Ala247Val) variant and a maternal c.783G > C (p. Gln261His) variant and both their parents are unaffected. However, the study did not conduct functional experimental validation. Our mRNA, protein and co-IP data provide preliminary functional evidence that p. Ala247Val may be clinically relevant when inherited with another deleterious SIL1 variant. Nevertheless, both variants identified in the present family remain classified as variants of uncertain significance, and additional genetic and functional evidence will be required for definitive pathogenic classification.

The ER is a central organelle of the endomembrane system in eukaryotic cells and plays a key role in maintaining protein homeostasis. Molecular chaperones such as SIL1 and BiP act as crucial molecules that promote proper protein folding, prevent protein aggregation and precipitation, and degrade misfolded proteins (Sun et al., 2021; Chen et al., 2020; Yan et al., 2011; He et al., 2022). As the nucleotide exchange factor for BiP, SIL1 regulates the ATPase cycle of BiP, participates in the binding and folding assembly of nascent polypeptide chains and promotes the dissociation of BiP from its substrate proteins (Inaguma et al., 2014; Ichhaporia and Hendershot, 2021; Riazuddin et al., 2009; Otero et al., 2010; Labisch et al., 2018). Previous studies have shown that Sil1 gene mutations can cause deletion or amino acid alterations in the C-terminal ER retention sequence of its protein, thereby blocking its interaction with the BiP protein. This will lead to impaired BiP function, incorrect folding of substrates and loss of substrate function (Horvers et al., 2013; Howes et al., 2012). In the present study, AlphaFold-based structural modeling suggested possible changes at the predicted SIL1-BiP interface, and coimmunoprecipitation showed reduced BiP pulldown signal for mutant SIL1 constructs, particularly the combined p. Asn190del + p. Ala247Val construct. These data support a possible effect of the variants on SIL1-BiP interaction but should not be interpreted as definitive proof of downstream ER dysfunction. We plan to establish disease-relevant models, including compound heterozygous mutant mouse or cell models, to investigate downstream effects on ER stress, proteostasis and neuronal development.

## Conclusion

In summary, this study identified novel compound heterozygous SIL1 variants (c.570_572delCAA and c.740C > T) in a MSS patient. Patient-derived B-LCLs showed reduced SIL1 mRNA and protein levels, and functional assays suggested that the variants may weaken SIL1-BiP interaction. These findings expand the SIL1 variant spectrum and provide preliminary functional support for the relevance of these variants to the MSS phenotype in this family. This work highlights the significance of focusing on SIL1 compound mutations in similar cases and lays the groundwork for further exploration of SIL1 and MSS.

## Data Availability

The raw data supporting the conclusions of this article will be made available by the authors, without undue reservation.
