# Supplementary material for: Novel compound heterozygous SIL1 variants associated with Marinesco-Sjögren syndrome in a Chinese family
Source: Front Genet. 2026 Aug 28;17:1795147. doi: 10.3389/fgene.2026.1795147 (PMC13561499; doi:10.3389/fgene.2026.1795147)
Supplement: Supplementary file 1 [file DataSheet1.docx]

**Figure S1.** **Effects of the SIL1 mutations on apoptosis and immunofluorescence colocalization analysis.** (**A**) Flow cytometry detection of apoptosis in the B-LCLs from normal, I:1, I:2 and II:1. n=3 independent experimental repeats. Statistic data were analyzed using one-way ANOVA. (**B**) Immunofluorescence colocalization analysis of wild type and mutant plasmids overexpressed in Hela cells.

**Figure S2.** **Displays the vibrational entropy, atomic fluctuation and deformation energies between wild type and mutant structures.** (**A**) Displays the Vibrational Entropy of mutant Ala247Val structure. A visual representation of the chain in which the mutation occurs is shown. Amino acids colored according to the vibrational entropy change upon mutation. Blue represents a rigidification of the structure and red indicates a gain in flexibility. 3D Visual representation of atomic fluctuation (**B**) and deformation energies (**C**) of wild type and mutant structure are shown. The magnitude of the fluctuation is represented by thin to thick tube colored blue (low), white (moderate) and red (high).
